# Research and Performance Evaluation of Low-Damage Plugging and Anti-Collapse Water-Based Drilling Fluid Gel System Suitable for Coalbed Methane Drilling

**DOI:** 10.3390/gels11070473

**Published:** 2025-06-20

**Authors:** Jian Li, Zhanglong Tan, Qian Jing, Wenbo Mei, Wenjie Shen, Lei Feng, Tengfei Dong, Zhaobing Hao

**Affiliations:** 1 Zhonglian Coalbed Methane (Shanxi) Co., Ltd., Taiyuan 030000, China; 15020050834@163.com (J.L.); fangyw_thzy@163.com (Z.T.); chensixu_zf@163.com (W.M.); 2 Provincial Technological Innovation Center for Co-Extraction of Three Gases, Taiyuan 030008, China; 15650163201@163.com (W.S.); duanshengnan1191393003@outlook.com (L.F.); 3 Engineering Technology Branch, CNOOC Energy Technology & Services Co., Ltd., Tianjin 300452, China; mic7512@163.com; 4 Zhonglian Coalbed Methane Co., Ltd., Beijing 100016, China; 5 Ministry of Education (MOE) Key Laboratory of Petroleum Engineering, College of Petroleum Engineering, China University of Petroleum (Beijing), Beijing 102249, China; dtf010019@cup.edu.cn

**Keywords:** coalbed methane drilling, reservoir protection, low-damage, water-based drilling fluid, gel system

## Abstract

Coalbed methane (CBM), a significant unconventional natural gas resource, holds a crucial position in China’s ongoing energy structure transformation. However, the inherent low permeability, high brittleness, and strong sensitivity of CBM reservoirs to drilling fluids often lead to severe formation damage during drilling operations, consequently impairing well productivity. To address these challenges, this study developed a novel low-damage, plugging, and anti-collapse water-based drilling fluid gel system (ACWD) specifically designed for coalbed methane drilling. Laboratory investigations demonstrate that the ACWD system exhibits superior overall performance. It exhibits stable rheological properties, with an initial API filtrate loss of 1.0 mL and a high-temperature, high-pressure (HTHP) filtrate loss of 4.4 mL after 16 h of hot rolling at 120 °C. It also demonstrates excellent static settling stability. The system effectively inhibits the hydration and swelling of clay and coal, significantly reducing the linear expansion of bentonite from 5.42 mm (in deionized water) to 1.05 mm, and achieving high shale rolling recovery rates (both exceeding 80%). Crucially, the ACWD system exhibits exceptional plugging performance, completely sealing simulated 400 µm fractures with zero filtrate loss at 5 MPa pressure. It also significantly reduces core damage, with an LS-C1 core damage rate of 7.73%, substantially lower than the 19.85% recorded for the control polymer system (LS-C2 core). Field application in the JX-1 well of the Ordos Basin further validated the system’s effectiveness in mitigating fluid loss, preventing wellbore instability, and enhancing drilling efficiency in complex coal formations. This study offers a promising, relatively environmentally friendly, and cost-effective drilling fluid solution for the safe and efficient development of coalbed methane resources.

## 1. Introduction

Amidst the global energy structure transformation and upgrading, and the growing demand for clean energy, coalbed methane (CBM), as a crucial unconventional natural gas resource, has seen its strategic importance and economic value increasingly highlighted [1,2]. China boasts abundant CBM resources, ranking as the world’s third-largest CBM resource holder, surpassed only by Russia and Canada. Its CBM resources are predominantly distributed in regions such as Shanxi, Shaanxi, Guizhou, and Sichuan. It is estimated that CBM resources at depths shallower than 2000 m amount to approximately 36.81 trillion cubic meters, indicating immense potential for exploration and development. This holds significant implications for ensuring national energy security, optimizing the energy mix, and reducing greenhouse gas emissions [3,4,5,6]. In recent years, while China has made notable progress in CBM exploration and development, with a continuous increase in the number of surface extraction wells and their overall production, the overall development level remains relatively low. Single-well production and recovery rates still lag significantly behind international advanced standards. Among these, technical bottlenecks in drilling engineering stand out as a critical factor hindering the efficient and economic development of CBM [7,8,9,10].

CBM reservoirs exhibit significant differences from conventional oil and gas reservoirs in terms of their petrophysical properties and occurrence characteristics [11,12]. Coal seams are primarily composed of organic matter, and their unique “matrix-cleat” dual-porosity system serves as the main space for CBM storage and migration. Within this system, the coal matrix possesses extremely fine pores, primarily storing CBM in an adsorbed state. Conversely, the cleat system (comprising face and butt cleats) forms the main seepage pathways for CBM once it desorbs. The degree of cleat development and connectivity directly dictates the CBM production capacity. CBM reservoirs generally possess the following characteristics [13,14,15,16,17]: Low Porosity and Permeability: The coal matrix exhibits extremely low permeability (0.001~0.1 mD), leading to an overall highly heterogeneous, low-permeability reservoir. High Brittleness and Fragility: Coal seams possess low compressive strength and a high brittleness index. During drilling, the wellbore coal rock is highly susceptible to fracturing and spalling under the combined effects of drilling tool disturbance and wellbore pressure fluctuations, consequently leading to wellbore instability and downhole complications. Low Mechanical Strength and Collapse Susceptibility: Coal seams, particularly certain tectonic and soft coal types, are often loosely cemented and exhibit poor structural integrity. This makes them highly prone to borehole shrinkage, collapse, or even stuck pipe incidents during drilling, severely compromising drilling safety and efficiency. Strong Sensitivity to Drilling Fluids: Coal seams are highly sensitive to external fluids. Firstly, the coal matrix and any clay minerals present (e.g., illite, kaolinite, montmorillonite) can hydrate and swell upon contact with water, thereby plugging cleat pathways and reducing permeability. Secondly, coal has a certain degree of hydrophilicity, meaning drilling fluid filtrate invasion can alter the coal’s wettability, consequently impacting CBM desorption and production. Furthermore, an unsuitable drilling fluid system may react chemically with inorganic salts within the coal seam, leading to precipitation and further plugging. During CBM drilling, the primary reservoir damage mechanisms encountered include the following [18,19,20]: (1) Capillary blockage caused by drilling fluid filtrate invasion into fractures/cleats. (2) Physical plugging due to the deposition of solid particles from the drilling fluid on pore and cleat surfaces. (3) Reduced pore connectivity resulting from the water absorption and swelling of coal and clay particles. (4) Differential pressure plugging caused by the deposition of a thick filter cake. (5) Permeability impairment due to the pronounced stress sensitivity of coal seams, where reservoir structures undergo permanent deformation once disturbed. The cumulative effect of reservoir damage directly leads to a decline in CBM well productivity, unsatisfactory post-drilling production results, and increased costs associated with subsequent stimulation treatments like unplugging and hydraulic fracturing. Consequently, the research and development of a novel drilling fluid system that offers low damage, anti-collapse, and swelling inhibition functionalities is critical for enhancing the efficiency and economic viability of CBM drilling operations.

Historically, conventional water-based drilling fluids, such as bentonite–polymer systems or high-salinity brine systems, have been widely used in coalbed methane (CBM) drilling. Although these systems are cost-effective and can maintain basic wellbore stability in relatively uncomplicated deep formations, they often suffer from severe fluid loss and cause irreversible permeability damage in unique, low-permeability, and highly sensitive CBM reservoirs [21,22,23,24]. For example, while clay-based systems are commonly used, the clay particles tend to disperse upon hydration, leading to significant hydration and swelling of coal and clay minerals. This can block pore throats and result in irreversible reservoir damage. Foam drilling fluids, though advantageous in terms of cuttings transport and reduced wellbore disturbance, are highly sensitive to temperature and pressure, making them difficult to control. In deep, high-temperature, and high-pressure CBM drilling, foam collapse often occurs, which reduces cuttings-carrying capacity and increases the risk of formation contamination. Moreover, foam systems have limited capability in sealing the complex, multi-scale fracture networks commonly present in coal reservoirs. To address these challenges, researchers both in China and abroad have proposed various low-damage drilling fluid technologies [25,26,27,28]. In recent years, there has been a growing body of research on low-damage, plugging, and anti-collapse drilling fluid gel systems [29,30,31]. These drilling fluids offer excellent tunable rheology, inflict minimal damage to coal seams, and provide advantages such as enhanced cuttings transport, the formation of an elastic plugging layer, and reduced filtrate loss at the well bottom. In particular, polymer-based gel systems possess viscoelasticity, allowing them to self-adapt to downhole environmental changes via a shear-recovery mechanism, thus offering excellent plugging capability and wellbore stability. Internationally, companies like Schlumberger have developed a series of nanogel and polymer plugging agent systems, which have been tested in shale gas and CBM drilling with positive field outcomes [32,33]. Domestically, petroleum universities and research institutes have also conducted extensive research into anti-collapse drilling fluid systems and composite materials for CBM drilling fluids, achieving substantial progress in permeability recovery tests [34,35]. Despite the significant progress made with low-damage drilling fluid systems, several critical challenges remain, including stability under high-temperature and high-pressure conditions, environmental compatibility of materials, field adaptability of formulation and injection processes, and effective cost control. Limitations such as inadequate stability under complex geological conditions (e.g., high salinity, high temperature and pressure) and insufficient universal sealing capability for multi-scale fractures in coal seams hinder their large-scale application. In particular, in the coalbed methane reservoirs of the LS block in the Ordos Basin—characterized by high fracture density, brittle structure, strong stress sensitivity, and susceptibility to drilling fluid-induced formation damage—the existing drilling fluid systems still fall short in simultaneously achieving multi-scale fracture sealing, strong inhibition of coal and clay swelling, minimization of reservoir damage, and thermal stability. As a result, they are unable to comprehensively and effectively address all the challenges encountered in complex coal seam drilling.

To address these multiple challenges—stemming from the well-developed fractures, fragile structure, strong stress sensitivity, and the susceptibility of coalbed methane (CBM) reservoirs to drilling fluid-induced damage—this study proposes a novel low-damage, plugging, and anti-collapse water-based gel drilling fluid system (ACWD). Unlike conventional single-function solutions, the ACWD system is uniquely designed to synergistically integrate excellent multi-scale fracture sealing capability, strong inhibition of coal and clay hydration, outstanding rheological stability under high temperatures, and minimal permeability damage to the reservoir. This study aims to offer a new low-damage, high-efficiency, and relatively environmentally friendly drilling fluid solution for CBM operations. Specifically, it addresses the complex issues of wellbore instability and reservoir damage commonly encountered during drilling in the LS block of the Ordos Basin. The ACWD system holds significant engineering value and practical relevance in enhancing China’s CBM exploration and development, reducing drilling costs, and improving single-well productivity.

## 2. Results and Discussion

### 2.1. Coal Seam Whole-Rock and Clay Mineral Composition Analysis

Table 1 and Table 2 present the whole-rock and clay mineral compositions of two coal seam samples obtained from distinct depths within the LS block. Both samples exhibited a high clay mineral content (exceeding 80% in each), characterized by predominant clay-cemented pores. The presence of numerous micropores and fine pore throats contributed to their overall low permeability. While clay minerals inherently possess some adsorption capacity and microporosity, their morphology and spatial arrangement significantly influence the overall pore structure. A significant transformation in clay mineral types was observed from the shallower core LS-C1 to the deeper core LS-C2: from predominantly kaolinite to predominantly illite and illite/smectite mixed layers. This transformation reflects a typical diagenetic trend. While kaolinite typically exhibits larger pores, illite and montmorillonite (which are particularly prone to swelling upon contact with water) can lead to pore blockage and reduced permeability.

### 2.2. Coal Seam Microstructure Analysis

**Figure 1:**
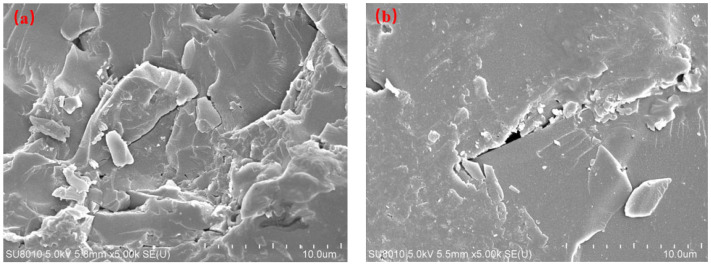
Scanning electron microscopy (SEM) images of coal rock from LS block. (**a**) Clay minerals; (**b**) Fracture structure.

Figure 1 presents scanning electron microscopy (SEM) images of coal rock from the LS block, illustrating its high degree of heterogeneity at the micro-scale. Figure 1a reveals numerous flaky or scaly particles of varying sizes and irregular shapes, identified as clay minerals such as kaolinite and illite, consistent with the analysis presented in Section 2.1. The matrix exhibits well-developed intergranular pores, organic pores, and micro-fractures; however, many are occluded by flaky detritus and cementing materials. Figure 1b shows fine, irregularly shaped mineral particles, while pervasive macro-fractures are observed, indicative of tectonic activity or stress release. In fractured regions and along fracture edges, porosity resulting from mineral dissolution or particle accumulation is evident. These pores and fractures collectively serve as the primary storage spaces for coalbed methane. Micropores primarily store gas via adsorption, whereas macropores and fractures hold gas in a free state and function as the principal pathways for fluid flow.

### 2.3. Evaluation of Basic Properties

This study evaluated the rheological and filtration performance of the prepared ACWD anti-collapse water-based drilling fluid gel system both before and after 16 h of hot rolling at 120 °C. The results are presented in Table 3. Both before and after 16 h of hot rolling at 120 °C, the ACWD system demonstrated stable rheological and filtration properties, with minimal changes in viscosity and shear stress. It exhibited “low viscosity and high shear” rheological characteristics. The initial API filtrate loss was low, and the filtrate loss after hot rolling only increased by 0.2 mL, with the high-temperature, high-pressure (HTHP) filtrate loss being merely 4.4 mL. This indicates that the ACWD system possesses a temperature resistance of up to 120 °C, fully satisfying the drilling requirements for the complex formations encountered in this block. In addition, the ACWD system consistently maintains a stable pH value, approximately 8.5 before hot rolling and around 8.0 afterward, which is attributed to the buffering capacity of its additives. This alkaline environment is crucial for effectively inhibiting the swelling of clays and coal formations. By limiting the hydration and dispersion of water-sensitive minerals, it contributes to the overall stability and anti-collapse performance of the system.

### 2.4. Evaluation of Settling Stability

Following the test methodology outlined in Section 4.3.2, a 1.40 g/cm^3^ ACWD anti-collapse water-based drilling fluid gel system was poured into an aging cell. This sample was then hot-rolled at 120 °C for 16 h in a roller oven. After subsequent static aging for 24, 48, 72, and 96 h, the densities of the upper and lower sections of the drilling fluid system were measured. The test results are presented in Table 4. The ACWD system exhibited a static settling coefficient (SF) of 0.500 after standing undisturbed for both 24 and 48 h. After 72 and 96 h of static settling, the SF values were merely 0.504 and 0.509, respectively. It is generally accepted that a system is considered stable when SF is between 0.50 and 0.53 (inclusive). An SF value greater than 0.53 indicates significant settling of the weighting agent or high-density solid phase, necessitating adjustments to the rheology or density. These results clearly demonstrate that the ACWD system possesses excellent static settling stability.

### 2.5. Evaluation of Inhibition Performance

When coalbed methane (CBM) wells in the LS block encounter carbonaceous mudstone sections, bedding planes and micro-fractures are well developed. Under the combined effects of high differential pressure and spontaneous imbibition, filtrate rapidly invades the formation, leading to wellbore instability. The Shihezi Formation, characterized by its high clay mineral content, exhibits significant swelling. Specifically, kaolinite undergoes extensive mudding upon contact with water, making the formation highly susceptible to disintegration upon water exposure. In accordance with the petroleum industry standard SY/T 5613-2016, “Test of drilling fluids—Test method of physical and chemical properties for shale [36],” carbonaceous mud shale samples from the LS block were selected for experimental investigation. The inhibitory performance of the developed ACWD plugging and anti-collapse drilling fluid system was then evaluated, using the rock sample’s expansion rate and rolling recovery rate as key indicators.

#### 2.5.1. Linear Expansion Experiment

**Figure 2:**
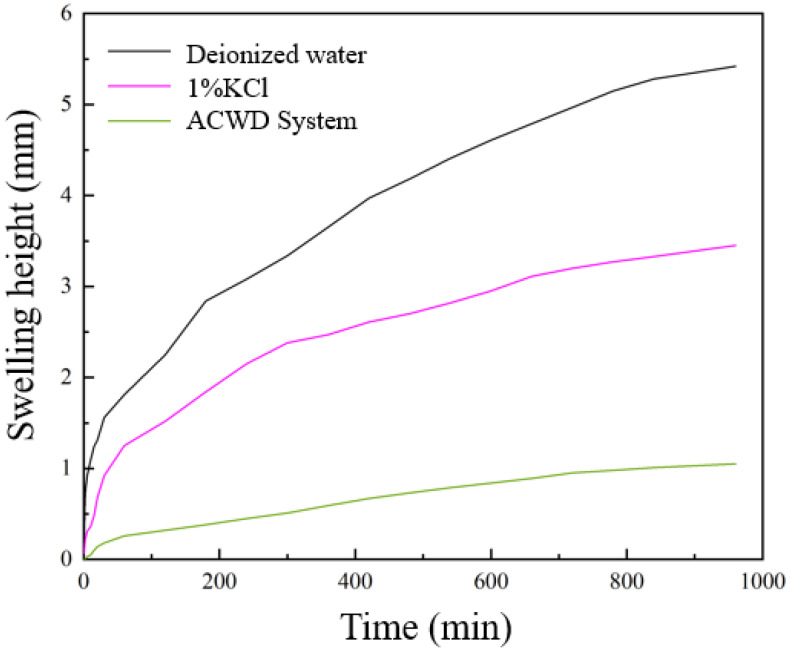
Rock sample linear expansion curves.

Linear expansion experiments were conducted on field-retrieved rock samples using deionized water, 1.0% KCl solution, and the prepared ACWD water-based drilling fluid gel system. The test results are presented in Figure 2. The rock sample exhibited the most significant expansion in deionized water, reaching a final height of 5.42 mm. This pronounced swelling is attributed to the strong osmotic and hydration effects that occur when deionized water interacts with clay minerals in the shales. Negatively charged clay particle surfaces adsorb water molecules, forming hydration films between layers, which causes the clay lattice to expand, manifesting macroscopically as an increase in the rock sample’s volume. In contrast, the expansion heights for the 1.0% KCl solution and the ACWD system were 3.45 mm and 1.05 mm, respectively. Compared to deionized water, K^+^ ions, with their smaller radius and lower hydration energy, can readily penetrate the interlayers of clay minerals. They achieve this by ion-exchanging with highly hydrating cations such as Na^+^ and Ca^2^^+^ located within the interlayer spaces. Furthermore, K^+^ ions bind more tightly to the clay lamellae, restricting water molecule entry and thereby inhibiting clay hydration swelling. Beyond K^+^ ions, the ACWD system incorporates additional cationic inhibitors and plugging agents. These agents can effectively adsorb onto and encapsulate clay particles, creating a physical barrier that prevents water intrusion. Moreover, they are capable of entering rock micro-fractures and pores, forming a dense filter cake or plugging layer that reduces permeability and minimizes water–clay contact. Overall, the results demonstrate that the developed ACWD plugging and anti-collapse water-based drilling fluid gel system effectively inhibits the hydration swelling of shale in this block, thereby achieving wellbore stability and preventing complex downhole incidents such as wellbore instability.

#### 2.5.2. Shale Rolling Recovery Experiment

**Figure 3:**
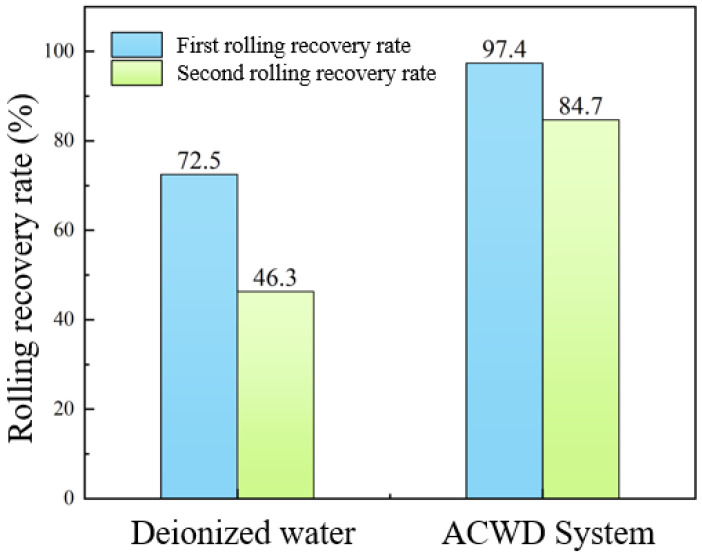
Rock sample rolling recovery rate.

Rolling recovery experiments were conducted on field-retrieved rock samples using both deionized water and the ACWD water-based drilling fluid gel system. The test results are presented in Figure 3. Deionized water yielded primary and secondary rolling recovery rates of 72.5% and 46.3%, respectively. In contrast, the ACWD drilling fluid system achieved significantly higher primary and secondary rolling recovery rates of 97.4% and 84.7%, respectively. This demonstrates not only that the ACWD system exhibits excellent initial inhibitory performance but also that the protective film it forms and its underlying inhibition mechanism are more stable and durable. This enables it to better withstand prolonged fluid erosion and mechanical disturbance, thereby mitigating further degradation of cuttings upon prolonged fluid exposure.

### 2.6. Evaluation of Contamination Resistance

#### 2.6.1. Cuttings Contamination Resistance

**Figure 4:**
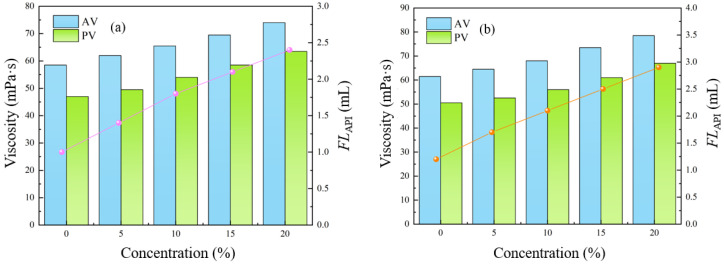
Rheological and filtration performance of ACWD system at varying cuttings concentrations: (**a**) BHR; (**b**) AHR.

To evaluate the resistance of the ACWD plugging and anti-collapse water-based drilling fluid system to cuttings contamination, field cuttings were added at concentrations of 5%, 10%, 15%, and 20%. The system was then hot-rolled at 120 °C for 16 h, and its properties were assessed before and after this treatment. The experimental results are presented in Table 5 and Figure 4. As the cuttings content increased, both apparent viscosity (AV) and plastic viscosity (PV) showed an upward trend. This indicates that the addition of cuttings increased the overall flow resistance of the drilling fluid. However, the magnitude of this increase was controllable, and the cuttings did not induce excessive dispersion of solids or other undesirable reactions within the drilling fluid. This demonstrates that the ACWD system possesses considerable resistance to contamination and thermal stability. Simultaneously, the static shear force also exhibited an increasing trend, which is beneficial for suspending cuttings and preventing their settling when the drilling fluid is static. Both the addition of cuttings and high-temperature aging contributed to maintaining or even enhancing the drilling fluid’s gel structure. The minimal difference between the initial and final gel strengths indicates good thixotropic properties of the drilling fluid, implying it is less prone to forming an overly rigid gel, which is advantageous for pump startup. Furthermore, while the incorporation of cuttings can somewhat influence the compactness of the filter cake, leading to a slight increase in filtrate loss, it is notable that even with a 20% cuttings content, the filtrate loss after aging was merely 2.9 mL. This indicates that the ACWD system is capable of forming a high-quality, low-permeability filter cake and possesses excellent inhibitory and plugging capabilities, effectively controlling liquid phase invasion into the formation, thereby contributing to wellbore stability.

#### 2.6.2. Salt Contamination Resistance

**Figure 5:**
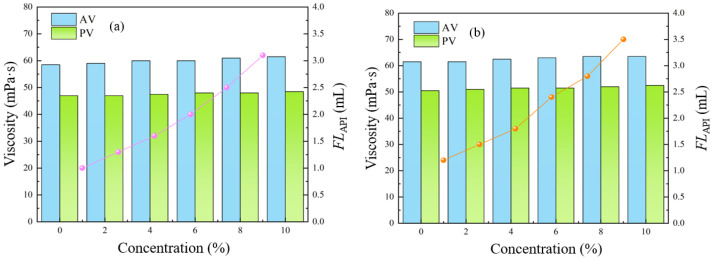
Rheological and filtration performance of ACWD system at varying CaCl_2_ concentrations: (**a**) BHR; (**b**) AHR.

We incorporated varying concentrations of CaCl_2_ into the developed ACWD plugging and anti-collapse water-based drilling fluid system and proceeded to evaluate its fundamental properties. The experimental results are presented in Table 6 and Figure 5. As the concentration of CaCl_2_ increased, the system’s density remained largely constant both before and after aging, indicating stable dissolution of CaCl_2_. Both apparent viscosity and plastic viscosity showed minimal increases before and after aging. The incorporation of CaCl_2_ and subsequent high-temperature aging contributed to maintaining or slightly enhancing the drilling fluid’s gel structure, which is advantageous for its suspension capacity. The small difference between initial and final gel strengths indicates that the system possesses good thixotropic and suspending properties. However, filtrate loss did show a slight increase. At a CaCl_2_ content of 10%, the filtrate loss was 3.5 mL. Importantly, this filtrate loss remains relatively low compared to other types of water-based drilling fluid systems. This demonstrates that the ACWD system forms a filter cake of consistently high quality with strong plugging capabilities, even under conditions of severe salt contamination and high temperature.

### 2.7. Evaluation of Plugging Performance

Coal seams inherently possess cleats and fractures, and insufficient drilling fluid plugging can lead to coal seam collapse and spalling. Furthermore, coal seams often contain significant quantities of gangue. Coal gangue, while possessing some brittleness and relatively low hardness, is also prone to fragmentation. When drilled through, this gangue readily fragments, contributing to formation collapse. Therefore, utilizing the developed ACWD water-based drilling fluid gel system, we evaluated the ACWD system’s plugging performance through sand bed and fracture plugging experiments.

#### 2.7.1. Sand Bed Plugging Experiment

**Figure 6:**
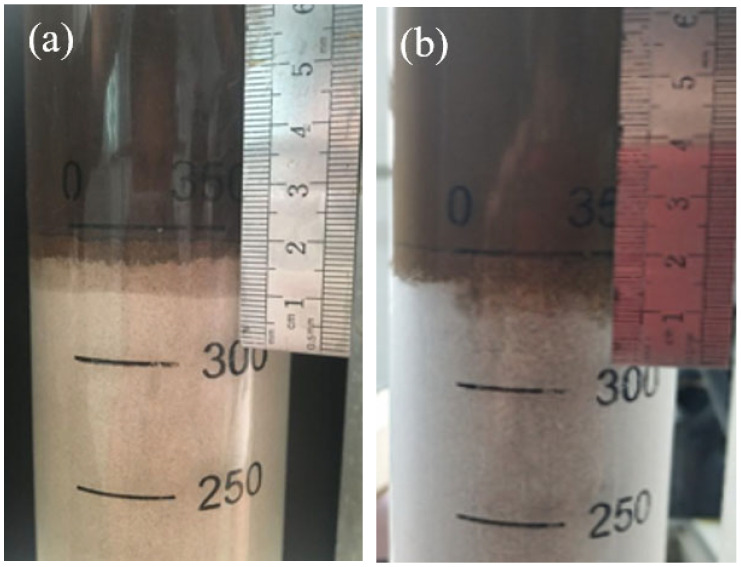
Sand bed plugging performance of ACWD water-based drilling fluid gel system: (**a**) 60–80 mesh; (**b**) 80–100 mesh.

The plugging performance of the ACWD drilling fluid system against permeable loss was evaluated using a sand bed filtration apparatus. Experimental results are detailed in Table 7 and Figure 6. The ACWD water-based drilling fluid system demonstrated an invasion depth of 9.4 mm into 60–80 mesh quartz sand and only 5.0 mm into 80–100 mesh quartz sand. Crucially, zero filtrate loss was observed in both instances, indicating the ACWD system’s excellent plugging performance.

#### 2.7.2. Fracture Plugging Experiment

The plugging capacity and pressure resistance of drilling fluids for 200 μm and 400 μm micro-fractures were evaluated using a multifunctional drilling fluid loss control simulation experimental apparatus. For comparative purposes, a polymer water-based drilling fluid system, PMWD, was also introduced (composition: 4% base slurry + 0.5% NaOH + 3.0% KCl + 0.2% XC + 0.2% PHPA + 2.5% SMP + 5.0% ester-based lubricant + 2.5% ultrafine calcium carbonate + barite). The experimental results are presented in Table 8. The developed ACWD water-based drilling fluid gel system was able to completely plug simulated 200 μm and 400 μm fractures, withstanding 5 MPa of pressure and demonstrating zero filtrate loss. This excellent sealing performance is primarily attributed to the combination of sulfonated asphalt and ultra-fine calcium carbonate, which together form a flexible yet robust sealing layer within the fractures, enabling effective adaptation to fractures of varying sizes. In contrast, the comparative PMWD polymer water-based drilling fluid system exhibited a filtrate loss of 8.7 mL when plugging 200 μm fractures. For 400 μm fractures, its filtrate loss progressively increased with rising pressure, leading to complete fluid loss at 5 MPa. These results clearly indicate that the developed ACWD system possesses excellent fracture plugging capabilities.

### 2.8. Evaluation of Lubrication Performance

**Figure 7:**
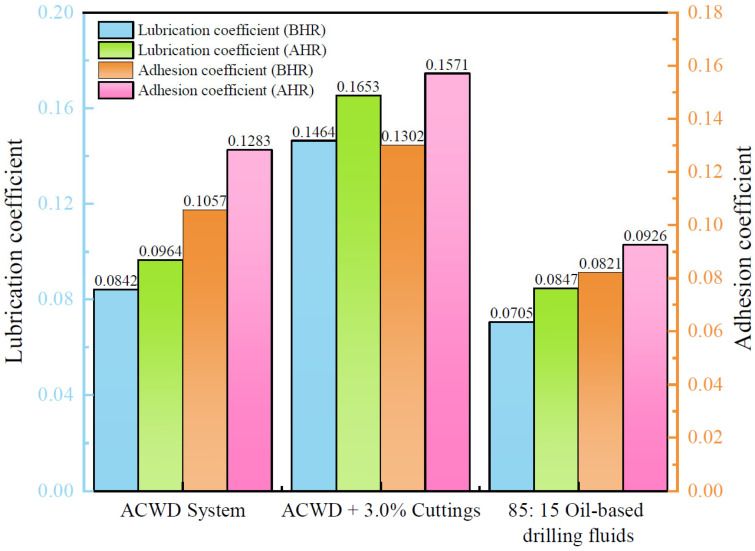
Lubrication performance evaluation of ACWD plugging and anti-collapse water-based drilling fluid gel system.

The extreme pressure (EP) lubrication coefficient and filter cake adhesion coefficient were measured using a Fann 21200 EP Lubricity Tester (Fann Instrument Company, Houston, TX, USA) and an NZ-3 Filter Cake Adhesion Coefficient Tester (Haitongda, Qingdao, China), respectively. Measurements were performed on the ACWD water-based drilling fluid system, the ACWD system contaminated with cuttings (formulated with 2.5% graphite + 1.0% erucamide), and an oil-based drilling fluid, all after aging at 120 °C for 16 h. The results are presented in Figure 7. The EP lubrication coefficients of the ACWD drilling fluid system were 0.0842 before hot rolling and 0.0964 after hot rolling. Although the lubrication coefficient slightly increased after aging, it remained within an acceptable range. However, with the addition of 3.0% cuttings, both the lubrication coefficient and filter cake adhesion coefficient of the ACWD system increased significantly. This indicates that cuttings contamination has a distinct negative impact on the lubricating performance and anti-sticking/pipe-sticking capabilities of the ACWD system. Therefore, in practical applications, close attention must be paid to solid phase control and the timely removal of cuttings. Compared with oil-based drilling fluids, the ACWD system shows relatively similar results in extreme pressure lubricity and anti-adhesion sticking resistance tests, although there is still a certain performance gap. This indicates that the ACWD system possesses inherent lubricating capability, primarily due to the presence of graphite and erucamide, which help reduce friction during drilling and protect both the wellbore and drilling tools.

### 2.9. Evaluation of Core Damage

We conducted core permeability tests on coal samples from the LS block using filtrates from both the formulated ACWD water-based gel drilling fluid system and the PMWD polymer water-based drilling fluid system. The core damage experiments were carried out under simulated formation pressure conditions, with a confining pressure of 10 MPa, a backpressure of 2 MPa, and a filtrate injection pressure of 1.5 MPa. During the experiment, permeability was measured using the constant flow rate displacement method, with a displacement rate set at 0.5 mL/min. The results, presented in Table 9, show that the core damage rates for coal samples LS-C1 and LS-C2 were 7.73% for the ACWD system and 19.85% for the PMWD system, respectively. The damage rate caused by the ACWD system was significantly lower than that of the PMWD system, indicating that the developed ACWD system provides superior protection to the reservoir.

### 2.10. Analysis of Coal Seam Wellbore Instability Mechanisms

**Figure 8:**
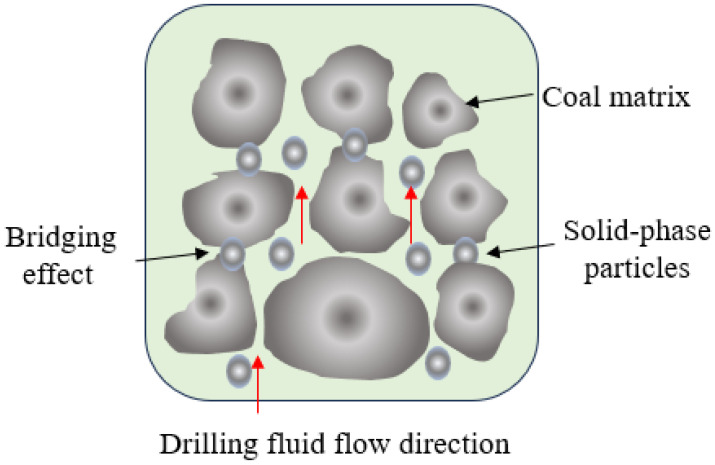
Schematic diagram of pore throat plugging by drilling fluid solid particles.

As highly fractured formations, coal seams are prone to wellbore instability during drilling operations. Their well-developed fracture and joint systems, coupled with external forces, primarily lead to wellbore collapse and hole shrinkage. This readily causes issues such as tripping resistance, pipe sticking, and pump choking, leading to downhole complications or the formation of “dog-leg” or “washout” wellbores, thus impacting normal coal seam drilling. During drilling, both bentonite particles in the drilling fluid and entrained drill cuttings can, with the flow of the drilling fluid, cause blockage of reservoir fractures, leading to reservoir damage, as illustrated in Figure 8. The more dispersed these solid particles are, the greater the reservoir damage, as fine particles can invade smaller micro-fractures through larger ones, thereby blocking the entire flow pathways within the reservoir and severely hindering subsequent coalbed methane drainage and production [37].

Experimental analysis of coal seams in the Ordos Basin’s LS block has revealed the prevalent development of two or more sets of nearly vertical natural fractures (face and butt cleats). These fractures significantly reduce the overall strength and integrity of the coal body, serving as preferential pathways for stress concentration and failure. The extensive development of cleats and micro-fractures in coal rock facilitates downhole fluid flow pathways, allowing easy drilling fluid invasion into the coal seams and extending deep into the formation along these micro-fractures. Such filtrate invasion further exacerbates fracture propagation and instability. Coal seams possess a complex pore structure, encompassing micropores, mesopores, macropores, and fractures, which collectively influence drilling fluid filtration behavior and the interaction between coal rock and fluids. Upon water-based drilling fluid filtrate invasion, water-sensitive clay minerals (e.g., montmorillonite, illite/smectite mixed layers) present in the shales absorb water and swell. This generates swelling stress, leading to a reduction in coal rock strength, an increase in volume, and subsequently, wellbore spalling or collapse. Therefore, these conditions necessitate higher demands on drilling fluid performance. The design of such fluids should prioritize robust plugging, low damage, strong inhibition, high lubricity, and wellbore strengthening capabilities. The drilling fluid should incorporate rigid and flexible plugging materials with a well-controlled particle size distribution (e.g., ultrafine calcium carbonate, sulfonated asphalt-based plugging agents) to effectively seal pore throats across various scales, from micro-fractures to larger fissures. This prevents deep invasion of drilling fluid filtrate, forms a tenacious filter cake, and enhances the overall pressure-bearing capacity of the wellbore. Highly effective inhibitors, such as potassium ions (e.g., potassium chloride), should be employed to reduce the hydration activity of clay minerals. Water activity can be lowered by increasing the salt concentration in the drilling fluid or by adding specific chemical agents, thereby minimizing the migration of water molecules into the formation. Furthermore, we incorporated graphite and erucamide to reduce frictional torque between the drilling tools and the wellbore. This minimizes mechanical scraping and disturbance of the wellbore by the drill string, protecting the integrity of the formed filter cake and preventing its degradation.

### 2.11. Field Application Case Study

The developed ACWD plugging and anti-collapse water-based drilling fluid gel system was successfully field-applied in the JX-1 well, a development well with a designed depth of 1823.6 m, located in the LS block of the Ordos Basin. Key drilling challenges in this well included severe, non-recoverable lost circulation and continuous seepage in the upper Shuangshi Formation, representing complex types of fluid loss. Moreover, substantial fluid loss risks were anticipated while drilling through the No. 4 + 5 and No. 8 + 9 coal seams of the Shanxi Formation. The horizontal sections, characterized by well-developed micro-fractures in the coal seams, were susceptible to fluid loss. Furthermore, drilling long horizontal coal sections necessitated a high anti-collapse performance. Specifically, these challenges often manifested as severe, non-recoverable lost circulation, continuous seepage through micro-fractures, cuttings bed accumulation in high dogleg severity sections, collapse of horizontal coal seams, and poor stability of interbedded coal layers. During actual drilling operations, the drilling fluid’s performance required timely adjustment and maintenance, based on real-time factors such as drilling rate and mud consumption, to ensure optimal cuttings carrying capacity, lost circulation control, and anti-collapse effectiveness.

**Figure 9:**
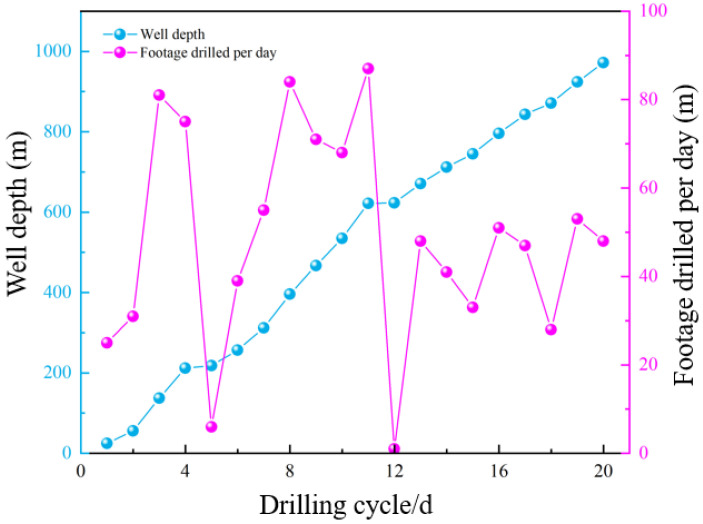
Cumulative and daily footage distribution for JX-1 well.

The basic properties of the ACWD drilling fluid system at different stages during the drilling of Well JX-1, including density and viscosity, are shown in Table 10. Throughout field application, the ACWD system maintained stable performance, with the overall fluid loss effectively controlled at around 3 mL, significantly reducing fluid loss during the drilling process. In formations prone to leakage and collapse, we promptly adjusted the dosages of sulfonated asphalt and KPAM, along with the drilling fluid density, to meet the specific drilling conditions of this block. The total drilling cycle for JX-1 well spanned 35.8 days. As depicted in Figure 9, which shows the cumulative and daily effective footage for this section, complex lost circulation (a combination of non-recoverable lost circulation and continuous seepage) occurred during drilling from the Shiqianfeng Formation to the Taiyuan Formation. For this well section, a composite drilling plan was adopted, integrating the ACWD drilling fluid system’s “drilling-while-controlling-loss” and “static plugging” capabilities. Compared with empirical data from adjacent wells, the ACWD drilling fluid system demonstrated remarkable fluid loss control in Well JX-1, significantly reducing drilling fluid loss circulation and substantially shortening lost circulation treatment time, thereby effectively preventing wellbore instability. Additionally, the system’s excellent rheological properties ensured strong cuttings-carrying capacity. As a result, the overall rate of penetration (ROP) increased by 24.7%, greatly enhancing drilling efficiency. No wellbore collapse, hole shrinkage, or other complex issues occurred in the coal seam section during the drilling process. The successful completion of JX-1 well proved the correctness of the ACWD drilling fluid system’s anti-collapse and lost circulation control theory, effectively resolving technical challenges related to fluid loss prevention, plugging, and anti-collapse during horizontal drilling of coalbed methane wells.

## 3. Conclusions

This study successfully developed and evaluated a novel low-damage, plugging, and anti-collapse water-based drilling fluid gel system (ACWD), designed to address the numerous challenges encountered during coalbed methane (CBM) drilling, particularly in low-permeability, highly brittle, collapse-prone, and drilling-fluid-sensitive coal reservoirs. The developed ACWD gel system effectively controls filtrate loss and forms a high-quality, thin, and flexible filter cake on the wellbore, which is crucial for minimizing fluid invasion into the fragile coal matrix. A key highlight of this system is its outstanding reservoir protection capability. It exerts a powerful inhibitory effect on the hydration and swelling of water-sensitive clay minerals and the coal matrix, significantly mitigating their volumetric expansion. Furthermore, the ACWD system demonstrates excellent plugging capabilities for both porous and fractured formations, effectively sealing micro-fractures and preventing deep fluid invasion. This is vital for maintaining wellbore stability and reducing permanent formation damage. The system also provides good lubricating performance, helping to reduce torque and drag during drilling, thereby minimizing mechanical damage to the wellbore. Compared to conventional drilling fluids and existing low-damage solutions, the ACWD gel system significantly reduces coal seam permeability damage, leading to enhanced post-drilling productivity. The ACWD system demonstrates superior performance compared to conventional drilling fluids and existing low-damage solutions. For instance, in fracture sealing evaluations, the ACWD system achieved complete sealing of 400 μm fractures with zero fluid loss, significantly outperforming the PMWD polymer system, which exhibited substantial fluid loss and full penetration under a pressure of 5 MPa. Moreover, the ACWD system caused an exceptionally low core damage rate of 7.73% on LS-C1 cores, far lower than the 19.85% recorded with the PMWD system. Its lubricity is also highly competitive, approaching that of an 85:15 oil-based drilling fluid, highlighting its comprehensive wellbore protection capability. These advantages contribute significantly to reducing non-productive time during coalbed methane drilling operations. Its successful field application has validated the system’s practical effectiveness in resolving complex drilling issues such as lost circulation and wellbore instability, consequently improving drilling efficiency and reducing operational risks. In conclusion, the low-damage, plugging, and anti-collapse water-based gel drilling fluid system developed in this study offers promising, efficient, and environmentally responsible drilling fluid solutions for coalbed methane (CBM) development. Compared with oil-based drilling fluids, the ACWD system demonstrates excellent environmental compatibility. Future work will focus on conducting a comprehensive environmental impact assessment of the ACWD system and its key components, aiming to provide a more solid basis for environmental compliance in support of its commercialization and large-scale application.

## 4. Materials and Methods

### 4.1. Materials and Instruments

The following materials were supplied by China Petroleum Engineering Technology Research Institute Co., Ltd. (Beijing, China): sodium-based bentonite (Na-BT), xanthan gum (XC), low-viscosity carboxymethyl cellulose (CMC-LV), potassium polyacrylate (KPAM), graphite, erucamide, sulfonated asphalt, non-ionic water-lock inhibitor FS-1, ultrafine calcium carbonate, barite, partially hydrolyzed polyacrylamide (PHPA), and sulfonated phenolic resin (SMP). Anhydrous sodium carbonate (Na_2_CO_3_), potassium chloride (KCl), calcium chloride (CaCl_2_), and sodium hydroxide (NaOH) were purchased from Sinopharm Chemical Reagent Co., Ltd. (Shanghai, China). Deionized water was prepared in-house. Core samples used in the experiments were obtained from the LS block in the Ordos Basin. All chemical reagents were used without further purification.

The instruments utilized in this study included the following: an X-ray diffractometer (PANalytical B.V., Almelo, The Netherlands), an SU8010 Cold Field Emission Scanning Electron Microscope (Hitachi, Tokyo, Japan), a ZNN-D6 six-speed rotational viscometer (Xinruide Petroleum Instrument Co., Qingdao, China), an XGRL-4 high-temperature roller oven, an NZ-3 filter cake adhesion coefficient tester, and an SD6A multi-unit API filter loss instrument (all from Haitongda, Qingdao, China). Additionally, a core flooding apparatus (Haian Petroleum Instrument, Nantong, China) and a Fann 21200 Extreme Pressure (EP) Lubricity Tester (Fann Instrument Company, Houston, TX, USA) were employed.

### 4.2. Preparation of Water-Based Drilling Fluid Gel System

A base slurry was prepared by adding 0.8 g of anhydrous Na_2_CO_3_ and 16 g of sodium-based bentonite to 400 mL of deionized water. This mixture was vigorously stirred at 8000 r/min for 20 min, followed by static aging in a sealed container for 24 h. Then, the following components were sequentially added to the base slurry: 0.5% (*w*/*w*, based on the total slurry mass; the same applies below) NaOH, 0.1% XC, 1.5% CMC-LV, 0.3% KPAM, 1.5% graphite, 0.5% erucamide, 3.0% sulfonated asphalt, 1.0% nonionic anti-water-blocking agent FS-1, and an appropriate amount of barite (for adjusting the system density). After the addition of each component, the mixture was thoroughly stirred at 8000 r/min for 5 min to ensure complete homogenization. The resulting formulation constitutes an anti-collapse water-based gel drilling fluid system suitable for coalbed methane drilling. The relevant drilling fluid materials and their functions are shown in Table 11. Given that the coal seams in this block exhibit well-developed micro-fractures and are highly susceptible to water-blocking damage, specific additives were included to ensure the prepared anti-collapse water-based drilling fluid system is optimally adapted for the CBM reservoirs of the LS block. Sulfonated asphalt and ultrafine calcium carbonate were specifically incorporated for their anti-collapse and plugging capabilities, while the water-lock inhibitor FS-1 was added to mitigate water-locking issues. A comprehensive performance evaluation of this system was then conducted. For research convenience, this system was designated as the ACWD system. Its final formulation is as follows: 4.0% base slurry, 0.5% NaOH, 0.1% XC, 1.5% CMC-LV, 0.3% KPAM, 1.5% graphite, 0.5% erucamide, 3.0% sulfonated asphalt, 1.0% non-ionic water-lock inhibitor FS-1, 5.0% KCl, 2.0% ultrafine calcium carbonate, and barite added to achieve a final density of 1.4 g/cm^3^.

### 4.3. Performance Evaluation Methods

#### 4.3.1. Basic Properties

In accordance with the petroleum and natural gas industry standard, GB/T 16783.1-2014 Field Testing of Drilling Fluids—Part 1: Water-Based Drilling Fluids [38], the fundamental properties of the ACWD system were evaluated at both room temperature and after 16 h of hot rolling at 120 °C. Rheological parameters, including Apparent Viscosity (AV), Plastic Viscosity (PV), and Yield Point (YP), were calculated as per Formulas (1)–(3) [39].

(1) AV=φ600/2

(2) PV=φ600−φ300

(3) YP=(2×φ300−φ600)/2

where *φ*600 and *φ*300 represent the viscometer readings at 600 revolutions per minute (RPM) and 300 RPM, respectively.

#### 4.3.2. Settling Stability

High-temperature static settling stability tests are employed to quantify the settling rate of weighting agents in drilling fluids under static conditions after pump shutdown at the wellsite. A 1.4 g/cm^3^ anti-collapse water-based drilling fluid gel system was subjected to hot rolling at 120 °C for 16 h. After hot rolling, the sample was removed and cooled to room temperature, then vigorously stirred for 30 min. It was subsequently poured into a 1000 mL measuring cylinder and allowed to stand undisturbed for 24, 48, 72, and 96 h, respectively. Samples were taken to measure the density of the upper drilling fluid at 2/5 of the cylinder’s height and the density of the lower drilling fluid at 4/5 of the cylinder’s height. Subsequently, the static settling coefficient (*SF*) was calculated using Formula (4).

(4) $$SF=\frac{\rho_{bottom}}{\rho_{top}+\rho_{bottom}}$$

where *ρ_top_* and *ρ_bottom_* represent the densities of the upper and lower sections of the drilling fluid, respectively.

#### 4.3.3. Inhibition Performance

The hydration and dispersion of clay minerals when exposed to external fluids are critical factors influencing wellbore stability in reservoirs. This study assesses the reservoir protection capability of the ACWD drilling fluid system by investigating its inhibitory effect on clay mineral swelling.

(1) Linear expansion experiment

Initially, 5–10 g of bentonite was accurately weighed and compacted at 10 MPa for 5 min. The resulting bentonite pellet was then placed on a linear expand meter. The expansion height of the pellet was continuously measured from the moment the drilling fluid was introduced. Measurements ceased after 24 h, yielding the linear expansion curve.

(2) Shale rolling recovery experiment

Thirty grams of field-sourced 6–10 mesh shale cuttings were accurately weighed and placed into an aging cell. A predetermined volume of the ACWD drilling fluid system was then added. The aging cell was subsequently subjected to hot rolling at a specified temperature for 16 h in a high-temperature roller oven. Following hot rolling, the cuttings were retrieved, dried, and their residual mass was determined. The rolling recovery rate (*R*) was calculated using the following Formula (5) [40]:

(5) $$R=\frac{m_{1}}{m_{2}}\times 100\%$$

where, *R*: rolling recovery rate (%); *m*_1_: mass of cuttings after hot rolling (g); *m*_2_: mass of cuttings before hot rolling (g).

#### 4.3.4. Contamination Resistance

During actual drilling operations, various soluble salts, formation fluids, and fine rock particles within the stratum can significantly alter drilling fluid properties. Such alterations, if not meeting required field performance, can lead to complex downhole incidents like stuck pipe and wellbore instability. Consequently, drilling fluid systems must exhibit robust contamination resistance. To this end, the developed ACWD plugging and anti-collapse water-based drilling fluid system was evaluated for its resistance to salt contamination (using CaCl_2_) and drill cuttings contamination (using field cuttings). Its rheological properties and filtration/filter cake performance were subsequently tested after high-temperature aging at 120 °C.

#### 4.3.5. Plugging Performance

The permeation plugging and fracture plugging performance of the ACWD water-based drilling fluid gel system was comprehensively evaluated through both sand bed plugging and fracture plugging experiments.

(1) Sand bed plugging experiment

The permeation plugging performance of the ACWD water-based drilling fluid gel system was assessed using 60–80 mesh and 80–100 mesh quartz sand as simulated porous media.

(2) Fracture plugging experiment

Using a multifunctional drilling fluid loss control evaluation instrument, the plugging effectiveness of the ACWD water-based drilling fluid gel system on simulated 200 µm and 400 µm fractures was evaluated under ambient temperature conditions.

#### 4.3.6. Lubrication Performance

At room temperature, the extreme pressure lubrication coefficient and filter cake adhesion coefficient were determined for two types of 1.4 g/cm^3^ ACWD anti-collapse water-based drilling fluid systems: the fresh system and the contaminated system (with 5.0% added lubricant). Both systems had undergone aging at 120 °C prior to testing. The measurements were performed using a Fann 21200 Extreme Pressure (EP) Lubricity Tester and an NZ-3 Filter Cake Adhesion Coefficient Tester.

#### 4.3.7. Core Damage

The core damage experiment primarily assesses the impact of the ACWD anti-collapse water-based drilling fluid gel system on core matrix permeability. Natural core samples from the designated block were chosen to evaluate the reservoir protection performance of the ACWD system, adhering to industry-standard evaluation methodologies. The core permeability damage rate (*Φ*) is calculated using Formula (6):

(6) $$\phi =\frac{K_{0}-K_{1}}{K_{0}}\times 100\%$$

where, *K*_0_: permeability before damage (10^−3^ μm^2^), *K*_1_: permeability after damage (10^−3^ μm^2^), *Φ*: core permeability damage rate (%).

## Figures and Tables

**Table 1:** Whole-rock mineral composition of coal samples from LS block.

Core Number	Well Depth/m	Mineral Content/%					
		Quartz	Barite	Calcite	Analcime	Siderite	Clay Minerals
LS-C1	1981.3	8.0	5.1	0.9	-	2.3	83.7
LS-C2	2139.0		5.7	-	4.4	-	89.9

**Table 2:** Clay mineral analysis of coal samples from LS block.

Core Number	Well Depth/m	Relative Content of Clay Minerals/%		
		I/S	It	K
LS-C1	1981.3	-	5.3	94.7
LS-C2	2139.0	16.4	83.6	-

Notes: I/S: illite–smectite mixed layer; It: illite; K: kaolinite.

**Table 3:** Basic properties of ACWD anti-collapse water-based drilling fluid gel system.

System	ρ /(g/cm^3^)	Condition	AV /(mPa·s)	PV /(mPa·s)	YP /(Pa)	Gel /(Pa/Pa)	FL_API_ /(mL)	FL_HTHP_ /(mL)	pH
ACWD	1.40	BHR	58.5	47.0	11.5	4.5/6.5	1.0	-	8.5
		AHR	61.5	50.5	11.0	5.0/7.5	1.2	4.4	8.0

Notes: BHR: before hot rolling; AHR: after hot rolling.

**Table 4:** Static settling stability of ACWD anti-collapse water-based drilling fluid gel system.

Settling Time/h	ρ_top_/(g/cm^3^)	ρ_bottom_/(g/cm^3^)	SF
24	1.40	1.40	0.500
48	1.40	1.40	0.500
72	1.39	1.41	0.504
96	1.39	1.44	0.509

**Table 5:** Rheological properties of ACWD system at varying cuttings concentrations.

Concentration/%	Condition	ρ/(g/cm^3^)	AV /(mPa·s)	PV /(mPa·s)	YP /(Pa)	Gel /(Pa/Pa)	FL_API_ /(mL)
0	BHR	1.40	58.5	47.0	11.5	4.5/6.5	1.0
	AHR	1.40	61.5	50.5	11.0	5.0/7.5	1.2
5	BHR	1.44	62.0	49.5	12.5	5.5/6.5	1.4
	AHR	1.44	64.5	52.5	12.0	6.0/7.0	1.7
10	BHR	1.48	65.5	54.0	11.5	6.0/7.5	1.8
	AHR	1.48	68.0	56.0	12.0	6.5/8.0	2.1
15	BHR	1.52	69.5	58.5	11.0	7.0/8.0	2.1
	AHR	1.52	73.5	61.0	12.5	7.0/8.0	2.5
20	BHR	1.57	74.0	63.5	10.5	7.5/9.0	2.4
	AHR	1.57	78.5	67.0	11.5	7.5/9.5	2.9

**Table 6:** Rheological properties of ACWD system at varying CaCl_2_ concentrations.

Concentration/%	Condition	ρ/(g/cm^3^)	AV /(mPa·s)	PV /(mPa·s)	YP /(Pa)	Gel /(Pa/Pa)	FL_API_ /(mL)
0	BHR	1.40	58.5	47.0	11.5	4.5/6.5	1.0
	AHR	1.40	61.5	50.5	11.0	5.0/7.5	1.2
2	BHR	1.40	59.0	47.0	12.0	4.5/6.5	1.3
	AHR	1.40	61.5	51.0	10.5	5.0/7.0	1.5
4	BHR	1.40	60.0	47.5	12.5	4.5/6.5	1.6
	AHR	1.40	62.5	51.5	11.0	5.0/7.5	1.8
6	BHR	1.40	60.0	48.0	12.0	5.0/7.0	2.0
	AHR	1.40	63.0	51.5	11.5	5.0/7.5	2.4
8	BHR	1.41	61.0	48.0	13.0	5.5/7.5	2.5
	AHR	1.41	63.5	52.0	11.5	6.0/7.5	2.8
10	BHR	1.41	61.5	48.5	13.0	6.0/7.5	3.1
	AHR	1.41	63.5	52.5	11.0	6.5/8.0	3.5

**Table 7:** Sand bed filtration evaluation results of ACWD water-based drilling fluid gel system.

System	Particle Size/m	Filtrate Invasion Depth/mm	Filtration Loss/mm
ACWD	60–80	9.4	0
	80–100	5.0	0

**Table 8:** Fracture plugging evaluation results of ACWD water-based drilling fluid gel system.

Systems	Fracture Size	Pressure-Hold Time/min	Plugging Status	
			Pressure/MPa	Filtration Loss/mm
ACWD	200 μm	3	0	0
		3	0.5	0
		3	1.0	0
		3	2.0	0
		3	3.0	0
		3	4.0	0
		3	5.0	0
	400 μm	3	0	0
		3	0.5	0
		3	1.0	0
		3	2.0	0
		3	3.0	0
		3	4.0	0
		3	5.0	0
PMWD	200 μm	3	0	0
		3	0.5	0
		3	1.0	0
		3	2.0	0
		3	3.0	0
		3	4.0	3.5
		3	5.0	8.7
	400 μm	3	0	0
		3	0.5	0
		3	1.0	0
		3	2.0	6.1
		3	3.0	15.5
		3	4.0	36.8
		3	5.0	penetration

**Table 9:** Permeability test results from core damage experiment.

Systems	Core Number	Length/mm	Diameter/mm	K_0_ (10^−3^ μm^2^)	K_1_ (10^−3^ μm^2^)	Core Damage Rate (%)
ACWD	LS-C1	50.24	25.47	3.351	3.092	7.73
PMWD	LS-C2	50.65	25.28	3.284	2.632	19.85

**Table 10:** Basic performance evaluation of ACWD water-based drilling fluid system at different stages in JX-1 well.

Section/m	ρ/(g/cm^3^)	AV /(mPa·s)	PV /(mPa·s)	YP /(Pa)	Gel /(Pa/Pa)	FL_API_ /(mL)
0~82	1.07~1.09	18.5~22.5	12.0~16.5	6.0~6.5	5~7/5~8	1.0~1.5
82~256	1.07~1.09	15.5~19.0	7.5~12.0	7.0~8.0	5~6/5~7	1.2~1.8
56~921	1.09~1.10	32.0~41.5	15.5~22.0	16.5~19.5	5~15/6~16	1.5~2.6
921~1728	1.09~1.10	28.5~37.5	19.0~25.0	9.5~12.5	5~15/6~16	1.5~3.1

**Table 11:** Components and functions of the ACWD water-based drilling fluid system.

Number	Designation	Concentration (%)	Function
1	Anhydrous Na_2_CO_3_	0.2	Softening water quality and buffering pH value
2	Na-BT	4.0	Used for preparing the base slurry of water-based drilling fluids
3	NaOH	0.5	Adjusts pH value and enhances the slurry-forming ability of the mud
4	Xanthan gum	0.1	Enhances the low-shear viscosity of the drilling fluid
5	CMC-LV	1.5	Reduces the fluid loss of the drilling fluid
6	KPAM	0.3	Inhibits shale hydration, swelling, and dispersion
7	Graphite	1.5	Improves the lubricity of the drilling fluid
8	Erucamide	0.5	Improves the lubricity of the drilling fluid
9	Sulfonated asphalt	3.0	Seals micro-fractures and micropores
10	Non-ionic water-lock inhibitor FS-1	1.0	Provides excellent anti-water-blocking performance
11	KCl	5.0	Inhibits hydration and swelling, and prevents clay dispersion
12	Ultrafine calcium carbonate	2.0	Exhibits excellent sealing performance
13	Barite	Added according to field requirements	Increases the density of the drilling fluid

## Data Availability

The data presented in this study are openly available in article.
